# Genome-Wide Analysis of MYB Genes in *Primulina eburnea* (Hance) and Identification of Members in Response to Drought Stress

**DOI:** 10.3390/ijms25010465

**Published:** 2023-12-29

**Authors:** Jie Zhang, Yi Zhang, Chen Feng

**Affiliations:** 1 Jiangxi Provincial Key Laboratory of Ex Situ Plant Conservation and Utilization, Lushan Botanical Garden, Chinese Academy of Sciences, Jiujiang 332900, China; zhangjie@lsbg.cn (J.Z.); zhangyi@lsbg.cn (Y.Z.); 2 School of Life Sciences, Nanchang University, Nanchang 330031, China

**Keywords:** *Primulina eburnea*, MYB transcription factor, gene family, drought stress

## Abstract

Due to periodic water deficiency in karst environments, *Primulina eburnea* experiences sporadic drought stress in its habitat. Despite being one of the largest gene families and functionally diverse in terms of plant growth and development, MYB transcription factors in *P. eburnea* have not been studied. Here, a total of 230 MYB genes were identified in *P. eburnea*, including 67 1R-MYB, 155 R2R3-MYB, six 3R-MYB, and two 4R-MYB genes. The R2R3-type *PebMYB* genes could be classified into 16 subgroups, while the remaining *PebMYB* genes (1R-MYB, 3R-MYB, and 4R-MYB genes) were divided into 10 subgroups. Notably, the results of the phylogenetic analysis were further supported by the motif and gene structure analysis, which showed that individuals in the same subgroup had comparable motif and structure organization. Additionally, gene duplication and synteny analyses were performed to better understand the evolution of *PebMYB* genes, and 291 pairs of segmental duplicated genes were found. Moreover, RNA-seq analysis revealed that the *PebMYB* genes could be divided into five groups based on their expression characteristics. Furthermore, 11 *PebMYB* genes that may be involved in drought stress response were identified through comparative analysis with *Arabidopsis thaliana*. Notably, seven of these genes (*PebMYB3*, *PebMYB13*, *PebMYB17*, *PebMYB51*, *PebMYB142*, *PebMYB69*, and *PebMYB95*) exhibited significant differences in expression between the control and drought stress treatments, suggesting that they may play important roles in drought stress response. These findings clarified the characteristics of the MYB gene family in *P. eburnea*, augmenting our comprehension of their potential roles in drought stress adaptation.

## 1. Introduction

Abiotic stresses primarily comprise factors such as drought stress, which can negatively impact plant growth and development and even lead to plant mortality [1,2]. Drought stress affects physiological, cellular, and molecular processes that are driven by a complex regulatory network in which transcription factors (TFs), kinases, and abscisic acid (ABA) play key roles in signal transduction [3,4]. Analysis of the genes involved in the drought stress response improves understanding of their function in molecular pathways, which ultimately paves the way for genetically modifying stress tolerance in crops such as rice and maize [5,6].

The MYB family is one of the largest gene families in plants and plays a crucial role in a variety of physiological functions, including signal transduction, primary and secondary metabolism, and stress responses [7,8,9]. MYB TFs in plants can be divided into four primary categories based on the arrangement and number of repetitions: 1R-MYB, 2R-MYB, 3R-MYB, and 4R-MYB with one to four repeats, respectively [10,11]. Each repeat forms a motif fold of the helix-turn-helix (HTH) structure with approximately 50 amino acids, and the regular interval of tryptophan plays a key role in maintaining the configuration of the HTH structure [12,13]. Furthermore, R2R3-MYB (2R-MYB) are predominantly present and widely investigated in plants such as *Arabidopsis thaliana*, rice, maize, and grape [14,15,16]. In addition, numerous research studies on model plants such as *Arabidopsis*, rice, and maize demonstrated that MYB genes are implicated in abiotic stress response [17,18,19].

*Primulina eburnea* (Hance) Yin Z. Wang is an evergreen perennial herb belonging to the family Gesneriaceae that is extensively distributed in southern China among karst landforms [20,21,22]. This contrasts sharply with the endemic distribution of other species in the genus *Primulina*. The characteristic habitat of *P. eburnea* in the karst landscape has high calcium content, thin soil, poor water holding capacity, and a shortage of nutrients, as well as a distribution of terrestrial islands [23,24,25]. Notably, *P. eburnea* stands out as one of the top genetic resources within the genus *Primulina* due to its excellent adaptability, wide distribution, and horticultural potential [26,27,28]. As a plant resource with broad development potential, *P. eburnea* was adapted to unpredictable drought stress in the karst landform. The abiotic stress response has been studied in many species. However, the mechanism of the drought stress response in *P. eburnea* has not been investigated.

In this study, we focused on the genome-wide identification of MYB genes in *P. eburnea* and investigated members related to drought stress response. A total of 230 genes were identified through a genome-wide survey, and phylogenetic analysis of the *PebMYB* genes was performed. To gain further insight, a combination of high-throughput expression analysis of genes in different tissues was performed, and the expression patterns of selected genes under drought stress treatments were measured using real-time quantitative PCR (qRT-PCR). Seven *PebMYB* genes involved in the drought stress response in *P. eburnea* were finally identified. The aims of this study are to lay a solid foundation for understanding the regulatory mechanism of MYB genes under stress conditions and to provide fresh perspectives on plant conservation in karst landforms.

## 2. Results

### 2.1. Identification and Characterization of MYB Genes in P. eburnea

A total of 230 *P. eburnea* MYB sequences were identified in *P. eburnea*, and the genes were labeled with the prefix *PebMYB* for clear designation (Supplemental Table S1, Figure S1). The *PebMYB* genes were unevenly distributed in the chromosome (Figure S1). Notably, 10 *PebMYB* genes (*PebMYB22*, *PebMYB32*, *PebMYB70*, *PebMYB78*, *PebMYB89*, *PebMYB153*, *PebMYB154*, *PebMYB155*, *PebMYB166*, and *PebMYB167*) were not located on any of the 18 assembled chromosomes (Supplemental Table S1). The average gene density across the entire genome of *P. eburnea* was approximately 39.14 genes per megabase (Mb), whereas the density of the *PebMYB* gene per Mb was notably lower at 0.28.

The R2R3-PebMYB proteins had an average length of 312.4 amino acid residues, ranging from 162 (PebMYB48) to 868 (PebMYB120). In contrast, the remaining PebMYB proteins displayed a broader span from 75 (PebMYB204, PebMYB218) to 1056 (PebMYB4R2), with an average length of 386. This considerable variation in amino acid length highlights the variation in the PebMYB proteins (Supplemental Table S1). A corresponding variance in relative molecular weight (Mw) was also observed in relation to amino acid length. For instance, the Mw of R2R3-PebMYB proteins ranged from 18,644.97 (PebMYB48) to 99,015.85 (PebMYB139), while that of the other PebMYB proteins ranged from 8645.75 (PebMYB218) to 117,733.6 (PebMYB4R2). Moreover, the isoelectric point (pI) exhibited a range from 4.92 (PebMYB98) to 10.2 (PebMYB145) in R2R3-PebMYB proteins and from 4.18 (PebMYB192) to 10.14 (PebMYB178) for the remaining types, highlighting the differences in the charge properties of the PebMYB proteins.

### 2.2. Phylogenetic Analysis and Classification of PebMYB Genes

**Figure 1:**
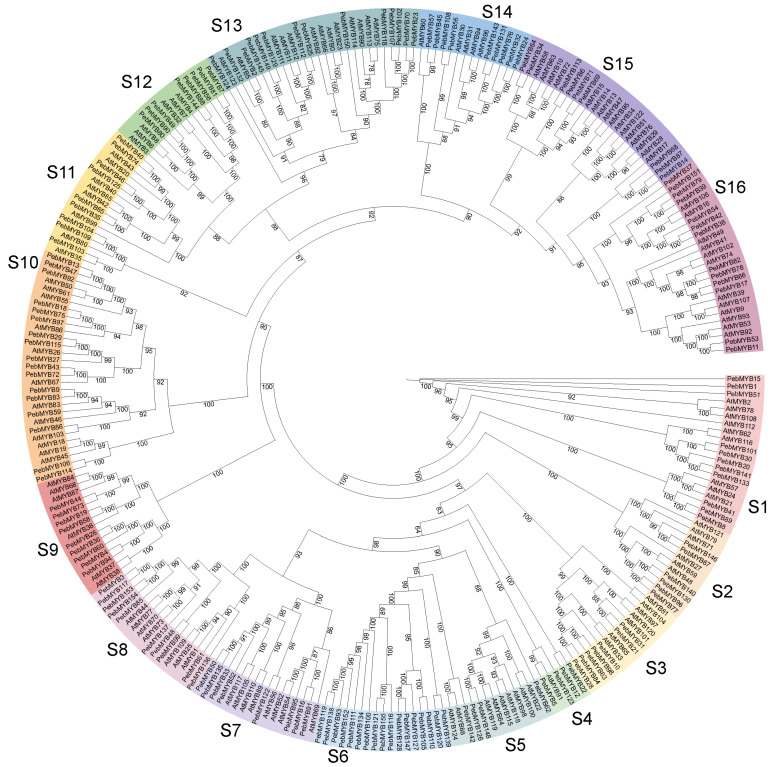
The phylogenetic relationship among R2R3-MYB genes in *Arabidopsis thaliana* and *Primulina eburnea*. The MYB genes were classified into 16 subgroups named S1 to S16 with different colors.

**Figure 2:**
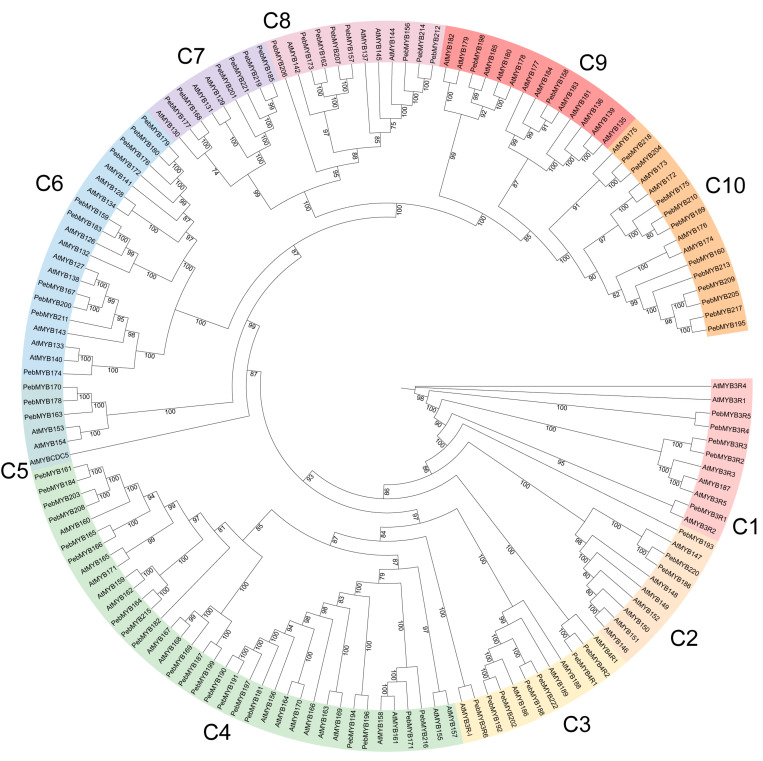
The phylogenetic tree of 1R-MYB, 3R-MYB, and 4R-MYB genes in *A. thaliana* and *P. eburnea*. The MYB genes were classified into 10 subgroups named C1 to C10 with different colors.

To investigate the phylogenetic relationship of the *PebMYB* and *AtMYB* genes, ML phylogenetic trees were constructed. The members of the phylogenetic tree were classified into several subgroups based on the topology of the tree and classifications in *A. thaliana* (Figure 1 and Figure 2).

In the phylogenetic tree of R2R3-MYB genes, 126 *A. thaliana* protein sequences and 155 *P. eburnea* sequences were classified into 16 subgroups and designated S1 to S16 (Figure 1). Notably, subgroups S4 and S5 were the smallest group and contained only four *PebMYB* genes. In contrast, subgroup S6 consisted of 17 *PebMYB* genes, making it the largest group. Interestingly, all the members in S6 belong to the *PebMYB* genes (Figure 1). Furthermore, the majority of subgroups contained *AtMYB* genes with known functions, which will be useful for studying the function of *PebMYB* genes. For example, members in S13 (*AtMYB66*, *AtMYB114*, *AtMYB90*, etc.) have been shown to play important roles in the biosynthesis of anthocyanin and flavonol, as well as seed germination in *A. thaliana*, suggesting that *PebMYB150* in S13 may also play a crucial role in anthocyanin biosynthesis [29,30,31].

The phylogenetic tree of the other MYB genes (1R-MYB, 3R-MYB, and 4R-MYB) contained 72 protein sequences from *A. thaliana* (64 1R-MYB, 5 3R-MYB, and 3 4R-MYB genes, respectively) and 75 protein sequences from *P. eburnea* (67 1R-MYB, 6 3R-MYB, and two 4R-MYB genes, respectively) (Figure 2). The members were classified into 10 categories (C1 to C10). C9, which was the smallest group, contained only two *PebMYB* genes, whereas the largest group, C4, included 20 members. Most *AtMYB* genes in C6 belonged to *CCA1*-*like* (*Circadian Clock Associated 1*), suggesting the likelihood that *PebMYB* genes in C6 may also function in circadian regulation. C2 contained *AtMYB* genes associated with *CPC*-*like* (*CAPRICE*), indicating that members of *PebMYB* genes in C2 could be involved in trichome formation and root development. C4 contained *A. thaliana* members of *TBP*-*like* (*telomeric DNA-binding protein*), which are known to play important roles in enhancing gene expression and cell development. Finally, C9 contained *R-R*-*type* MYB-like genes, while C10 contained *I-Box-Binding*-*like* genes from *A. thaliana* [16].

### 2.3. Motif Analysis and Gene Structure

**Figure 3:**
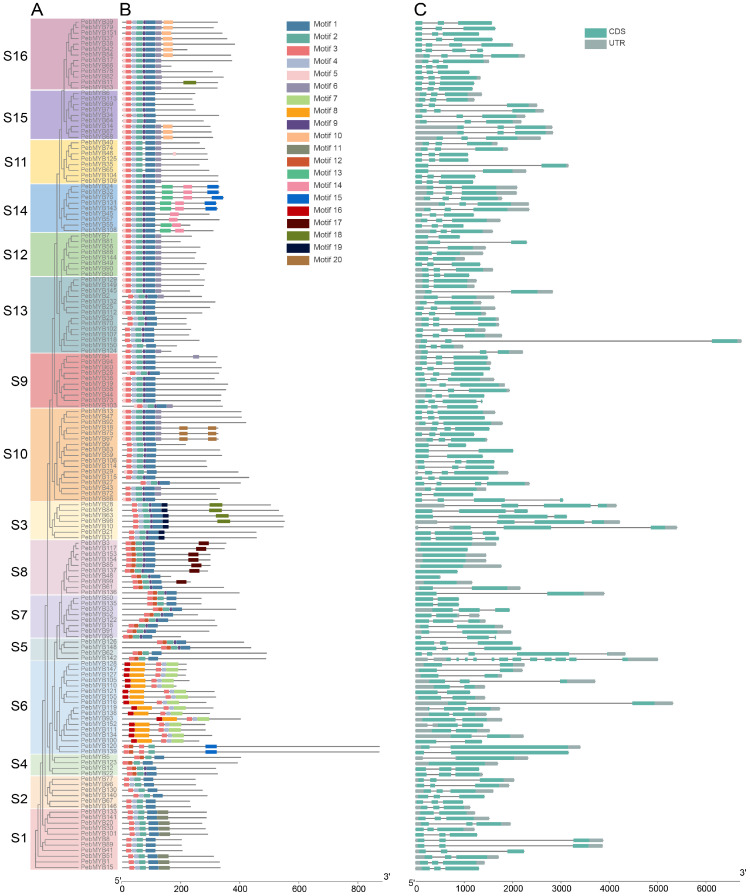
The phylogenetic tree, conserved motifs, and gene structure of R2R3-MYB genes in *P. eburnea*. (**A**) The ML phylogenetic tree of *PebMYB* genes grouped into 16 subgroups designated S1 to S16. (**B**) The conserved motif structure of *PebMYB* genes. (**C**) The gene structure of *PebMYB* genes. The grey bar indicates the untranslated region (UTR), whereas the cyan bar indicates the CDS. The x-axis in (**B**,**C**) indicates the lengths of proteins and genes, respectively.

**Figure 4:**
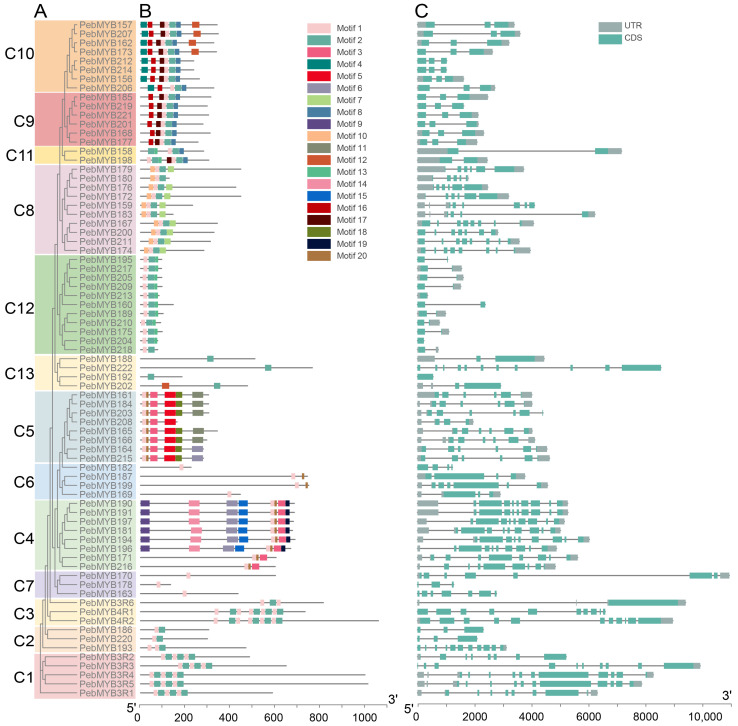
The phylogenetic tree, conserved protein motifs, and gene structure of 1R-MYB, 3R-MYB, and 4R-MYB genes of *P. eburnea*. (**A**) The ML phylogenetic tree of *PebMYB* genes grouped into 13 subgroups designated C1 to C13. (**B**,**C**) Conserved motifs and gene structure of *PebMYB* genes.

ML phylogenetic trees of 155 *R2R3-PebMYB* and 75 remaining *PebMYB* genes were constructed (Figure 3 and Figure 4). The topology of the phylogenetic tree was similar to the grouping pattern observed in the phylogenetic analysis in Figure 1 and Figure 2, confirming the reliability of the phylogenetic analysis and classification of *PebMYB* genes. Furthermore, the presence of similar motif compositions within the same subgroup further supported the grouping results.

To gain a comprehensive understanding of the conserved domains in *PebMYB* genes, motif analysis was performed by the MEME program with a parameter of up to 20 motifs found (Figure 3B and Figure 4B). The most common conserved motifs identified in the motif analysis of R2R3-MYB genes were Motif 1, Motif 2, Motif 3, and Motif 4, which consisted of 34, 22, 18, and 15 amino acids, respectively. The R2 repeat was composed of Motif 3, Motif 4, and Motif 2, while Motif 1 corresponded to the R3 repeat (Supplementary Figure S2A, Table S2). The HTH (helix-turn-helix) structure was formed by regularly spaced tryptophan residues (arrows in Figure S2A), which was consistent with previous studies in other species [32,33].

With only a few exceptions in S5, S6, and S7, the length of the R2R3 domain within the majority of *PebMYB* genes ranged from 10 to 150 amino acids (Figure 3B). Notably, Motif 1, Motif 2, and Motif 4 were either partially or completely absent in members of S4 and S6. This pattern was particularly evident in all 17 members of S6, which lacked either Motif 1 or Motif 2, separating them from other subgroups. These findings were similar to those found in Chinese jujube and upland cotton, indicating that similar motif compositions are features of the same subgroup and implying potential functional commonalities among members of the same subgroup [34,35]. The analysis of gene structures further revealed variations among subgroups (Figure 3C). For example, the majority of the R2R3-MYB genes in *P. eburnea* had two (40/155) or three (96/155) exons, only one gene (*PebMYB*62) had five exons, and one (*PebMYB*142) had 12 exons. Interestingly, these two genes belonged to S5. In addition, most members of S8 contained only one exon.

In the motif analysis of 1R-MYB, 3R-MYB, and 4R-MYB genes, two predominant motifs (Motif 1 and Motif 2) were identified. Motif 1 consisted of 18 amino acids and was composed of R2 MYB repeats, whereas Motif 2 contained 29 amino acids and was composed of both R1 and R3 repeats (Supplementary Figure S2B, Table S3). Motif 1 existed in most of the 1R-MYB, 3R-MYB, and 4R-MYB genes except for the members in C13, which distinguished them from the other groups. Regarding gene structure, approximately half of the genes (36 out of 75 genes) contained one to three exons, while 19 out of 75 genes exhibited five to seven exons. Notably, five genes contained as many as 11 exons. These findings highlighted a greater variation in gene structure and motif composition among 1R-MYB, 3R-MYB, and 4R-MYB genes compared to R2R3-MYB genes.

### 2.4. Gene Duplications and Synteny Analysis of PebMYB Genes

Gene duplication events play a crucial role in the diversity and evolution of gene families. To further understand the evolution and expansion of the *PebMYB* genes, a duplication analysis was performed. The analysis revealed a total of 291 pairs of segmental duplicated genes, including 176 *PebMYB* genes (Supplemental Table S4). The nonsynonymous and synonymous substitution ratios (Ka/Ks) values of all segmental duplicated gene pairs were significantly less than 1, suggesting that purifying selection may have played an important role in the evolution of the *PebMYB* genes. Interestingly, only 3 pairs of tandem duplicated *PebMYB* genes (*PebMYB147*, *PebMYB195*, and *PebMYB209*) were found.

**Figure 5:**
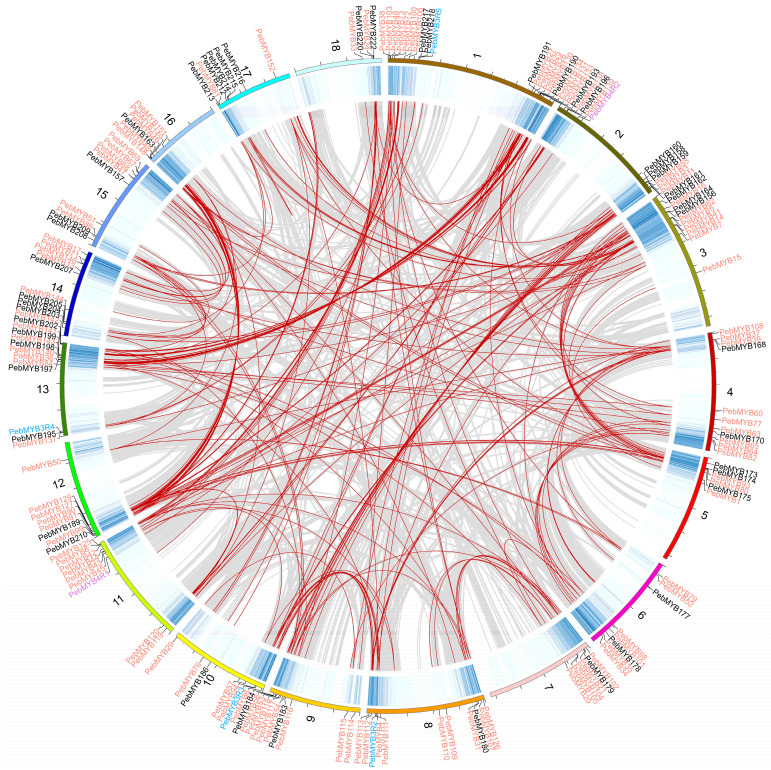
Duplication analysis of *PebMYB* genes. The heatmap indicates gene density. Grey lines indicate duplications of all genes, while duplications of *PebMYB* genes are highlighted by red lines. The black and grey scales on chromosomes indicate 10 Mb and 5 Mb, respectively. The 1R-MYB, 2R-MYB, 3R-MYB, and 4R-MYB genes are listed on chromosomes with black, salmon, blue, and purple colors.

Most *PebMYB* genes were located at the ends of chromosomes and were distributed unevenly across these regions (Figure 5 and Figure S1). Additionally, gene duplications were found in all chromosomes (Figure 5).

Synteny analysis of MYB genes between *P. eburnea* and two species (*A. thaliana* and *Oryza sativa*) revealed 234 orthologous gene pairs between *P. eburnea* and *A. thaliana*, whereas 35 orthologous gene pairs were found between *P. eburnea* and *O. sativa* (Figure S3). The synteny analysis results showed 26 collinear gene pairs across the three species, indicating that 107 collinear pairs between *A. thaliana* and *P. eburnea* no longer existed between *P. eburnea* and *O. sativa* (Supplemental Table S5).

### 2.5. Expression Profiles of PebMYB Genes in Different Tissues

**Figure 6:**
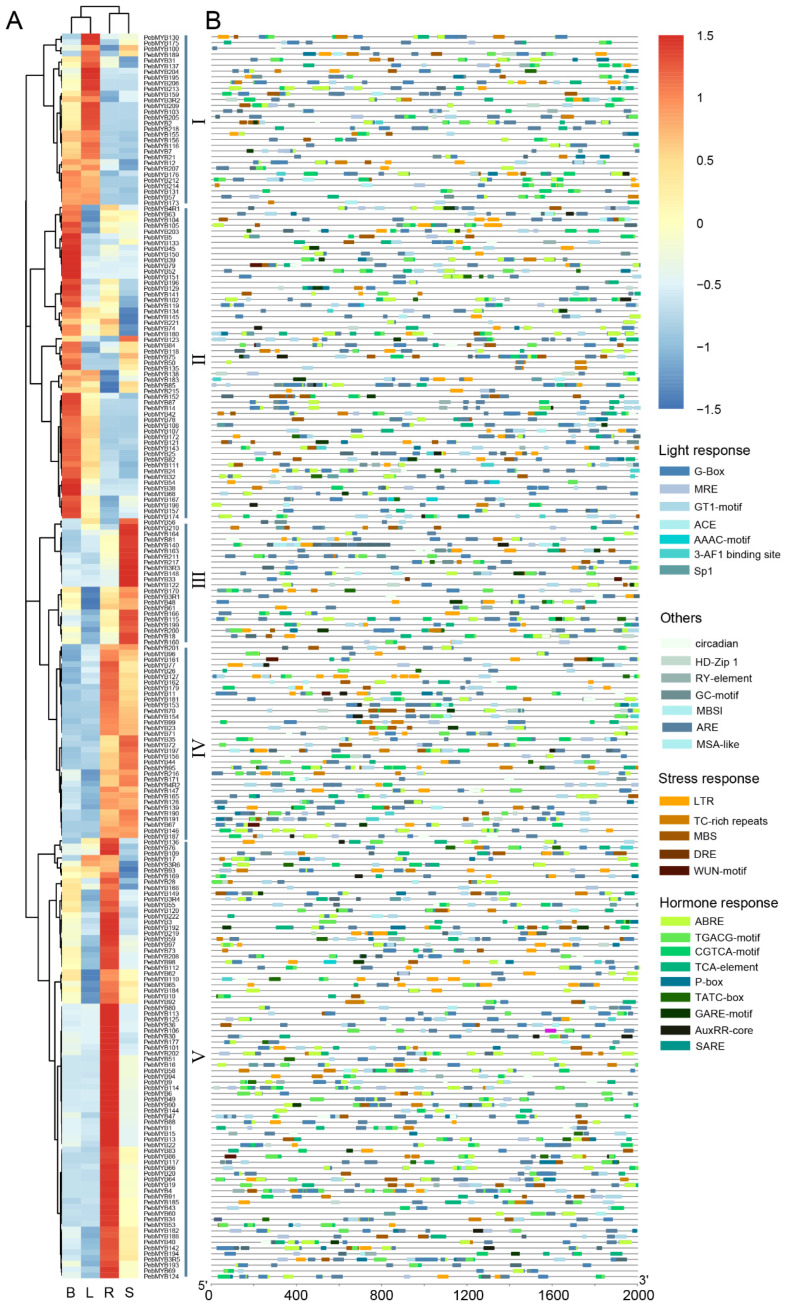
Expression pattern and promoter analysis of *PebMYB* genes. (**A**) The expression pattern of *PebMYB* genes in four different tissues (B, L, R, and S indicate bud, leaf, root, and stem, respectively) based on the RNA-seq data. (**B**) *Cis*-elements in the 2000 bp promoter region of *PebMYB* genes.

The expression patterns of *PebMYB* genes in stems, roots, buds, and leaves of *P. eburnea* were studied using RNA-seq data. Twelve *PebMYB* genes that showed no expression in any of the four tissues were excluded from the expression analysis. The remaining genes could be divided into five groups (I to V) based on their expression profiles. In group I, 30 *PebMYB* genes were highly expressed in leaf tissue, while 55 genes in group II were predominantly expressed in buds. The members of groups III and V (22 and 77 genes, respectively) showed high expression levels in the stem and root, respectively. Furthermore, the members of group IV (34 genes) showed high expression in both root and stem tissues (Figure 6A, Supplemental Table S6).

Furthermore, the *cis*-elements present in the promoters of *PebMYB* genes were analyzed to gain insights into their regulatory mechanisms. The *cis*-elements were primarily divided into four groups based on their functions: light response, stress response, hormone response, and others (Figure 6B, Supplemental Table S7). Notably, the hormone response and light response *cis*-elements were particularly abundant among the analyzed genes.

To verify the expression profiles from the RNA-seq data, several genes with high expression levels (fragments per kilobase of exon model per million mapped fragments, FPKM > 2) in at least one tissue were randomly selected, and the expression levels were determined by qRT-PCR. The relative expression levels of the chosen genes (11 genes) determined by qRT-PCR were consistent with the trends observed in the RNA-seq data, further supporting the reliability and accuracy of the RNA-seq results (Supplementary Figure S4, Table S6).

### 2.6. Expression Analysis of PebMYB in Drought Stress

MYB genes play a variety of roles, including anthocyanin biosynthesis, drought stress response, and many other functions. This notion was supported by the results of the promoter analysis, which highlighted numerous *PebMYB* genes associated with the stress response (Figure 6B). To investigate the role of *PebMYB* genes in the drought stress response, an experiment involving drought stress treatment was carried out. We conducted a homology search with the genome of *A. thaliana* using the protein sequences of 230 *PebMYB* genes. Based on functional annotation of the homologous genes in *A. thaliana* and their phylogenetic relationships, 11 *PebMYB* genes were selected for further investigation. The relative expression levels of the selected genes were determined by qRT-PCR at different developmental stages (ST0, ST1, and ST2).

**Figure 7:**
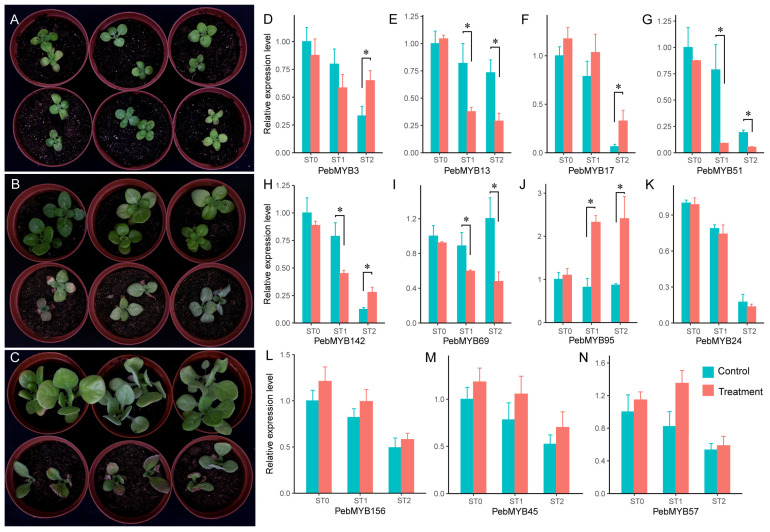
Expression analysis of selected *PebMYB* genes between the control and drought stress treatments. (**A**–**C**) The top three pots in (**A**–**C**) represent the control group, while the bottom three pots are the treatment group. The seedlings of *P. eburnea* before (**A**) and after drought stress treatment for one (**B**) and two months (**C**), respectively. (**D**–**N**) The relative expression levels of 11 selected *PebMYB* genes. ST0, ST1, and ST2 on the *x*-axis represent the seedling stages in (**A**–**C**), respectively. Asterisks above the columns show statistically significant differences at *p* < 0.05.

Among these selected genes, *PebMYB3* exhibited homology to *AtMYB44* (*AT5G67300*) and *AtMYB77* (*AT3G50060*), which is recognized for their involvement in mediating crosstalk between different signaling pathways in response to drought stress. Notably, these pathways included the ABA, auxin, salicylic acid (SA), and methyl jasmonate (MeJA) regulatory pathways, ultimately activating genes to prevent reactive oxygen species (ROS) accumulation [36,37,38]. Differential expression was observed between the control and treatment groups after treatment for 2 months (ST2 in Figure 7D). *PebMYB13*, along with its homologous gene *AT1G09540* (*AtMYB61*), was selected due to its role in processes such as stoma movement, root development, and seed germination. Interestingly, we noticed that the expression levels were significantly decreased in the treatment group compared to the control group in ST1 and ST2 (Figure 7E). In *A. thaliana*, a high expression level of *AtMYB61* could reduce stomatal aperture and stomatal conductance [39]. Likewise, *PebMYB17*, a homologous gene of *AT4G28110* (*AtMYB41*, function in suberin synthesis and assembly), was expressed significantly differential expression in ST2 (Figure 7F) [40,41]. *AT2G47190* (*AtMYB2*) and *AT3G06490* (*AtMYB108*) are homologous genes of *PebMYB51* that play crucial roles in response to drought and salt stress and regulate filament elongation and anther dehiscence through the jasmonic acid (JA) and gibberellic acid (GA) regulatory pathways [42,43]. The expression of *PebMYB51* displayed a rapid decline in the treatment group (Figure 7G). As the homologous gene of *PebMYB142*, *AT2G02820* (*AtMYB88*) is responsible for limiting cell division in the stomatal lineage and promoting stomatal closure in response to abiotic stress through the ABA regulatory pathway [44]. Furthermore, *AT3G23250* (*AtMYB15*, homologous to *PebMYB69*) was involved in responding to various stresses via ABA biosynthesis and signaling in *A. thaliana*. *PebMYB95* was homologous to *AT1G17950* (*AtMYB52*), which played crucial roles in cell wall architecture formation and affected ABA biosynthesis and response in *A. thaliana* [45]. In *P. eburnea*, the relative expression of *PebMYB142*, *PebMYB69*, and *PebMYB95* significantly differed between the treatment and control groups in ST1 and ST2 (Figure 7H–J and Figure S5).

*PebMYB45* and *PebMYB57* shared homology with *AT1G08810* (*AtMYB60*), a gene known for its involvement in the drought stress response, and were specifically expressed in guard cells and promoted stomatal opening when highly expressed [46,47]. However, the relative expression levels of *PebMYB45* and *PebMYB57* showed no significant difference between the control and treatment groups in *P. eburnea* (Figure 7M,N). Similarly, no significant differences were observed for *PebMYB156*, which is a homologous gene of *AT5G56840* with functions related to dehydration stress memory and sugar metabolism (Figure 7L) [48,49]. The homologous genes of *PebMYB24*, *AT3G47600* (*AtMYB94*), and *AT5G62470* (*AtMYB96*) played important roles in cuticular wax biosynthesis and accumulation in response to drought stress. However, no significant differences in expression were found between the control and treatment groups (Figure 7K) [50,51].

These results suggest that seven *PebMYB* genes (*PebMYB3*, *PebMYB13*, *PebMYB17*, *PebMYB51*, *PebMYB142*, *PebMYB69*, and *PebMYB95*) likely play important roles in the drought stress response in *P. eburnea*. However, it appears that *PebMYB45*, *PebMYB57*, *PebMYB24*, and *PebMYB156* do not respond to drought stress, as observed in *A. thaliana*. Further studies are needed to validate these findings and to elucidate the specific functions of these genes in the molecular mechanisms underlying drought stress adaptation in *P. eburnea*.

### 2.7. The Potential Co-Expression Network between PebMYBs and Other TFs

MYB genes could cooperate with other TFs to regulate their expression. To investigate the cooperative interactions, we calculated the PCC between *PebMYB* genes and other TFs across four tissues (Supplementary Table S8). A total of 2359 TFs from 48 families were predicted in this study using a cutoff PCC value greater than 0.95. These TFs were used to construct an interaction network (Figure S6). Among the interacting TFs, members from the AP2/ERF, C2H2, and WRKY families displayed prominent TFs that interact with *PebMYB* genes (Figure S6A). Further investigation revealed interesting patterns of interaction clustering in the network. For example, a notable cluster comprised more than 30 R2R3-type *PebMYB* genes, forming a complex network (Figure S6B). This observation suggested that developmental processes may be regulated by an intriguing network of multiple interacting partners. The interaction network of *PebMYB* genes and other TFs provided useful information for further investigation of the interactions between *PebMYB* genes and other TFs.

## 3. Discussion

*P. eburnea* is a promising candidate for development into ornamental plants, primarily due to its unique flower shape and colors [27,45]. Unlike the other species in the genus *Primulina*, which have limited endemic distributions, *P. eburnea* is widespread across several provinces in southern China, including diverse landscapes such as the Danxia and karst landforms. In addition, the natural soil conditions of its habitat do not retain water for extended periods, resulting in aperiodic drought stress. However, little is known about how *P. eburnea* adapts and responds to unpredictable drought stress. Unraveling the mechanisms by which *P. eburnea* adapts to the challenges of drought stress has important implications. Not only does it enrich our knowledge of how this species responds to drought conditions, but it also lays a foundation for advancing its conservation.

The MYB gene family is involved in regulating various biological activities, such as signal transduction, anthocyanin biosynthesis, and abiotic stress response [9,11,52]. However, comprehensive studies on the MYB gene family in *P. eburnea* have yet to be conducted. Here, we identified 230 *PebMYB* genes in *P. eburnea*. This number was greater than those found in *A. thaliana* (196) and *Populus trichocarpa* (197) but less than those in *Primulina swinglei* (264) and *Brassica rapa* (293) [9,53]. The reason may be the differential rates of gene family contraction or expansion among different lineages [54].

In the majority of subgroups, the number of *PebMYB* genes closely followed that of *AtMYB* genes, with a few exceptions. For instance, there was only one *AtMYB* gene in subgroup S4 while harboring four *PebMYB* genes. Similarly, S10 exhibited a comparable pattern with 11 *AtMYB* genes and 17 *PebMYB* genes. This gene expansion may be responsible for the gene duplication and differentiation of gene function [55]. For instance, *AtMYB91* in S4 is known to be involved in the specification of the leaf proximodistal axis [56]. Similar observations were found in S10, where members (*AtMYB103*, *AtMYB26*, etc.) contribute to lignin biosynthesis and stamen development [57,58]. Conversely, gene contraction was also found in certain groups. For instance, nine *AtMYB* genes in S5 coexisted with four *PebMYB* genes, while S15 consisted of 16 *AtMYB* genes and nine *PebMYB* genes (Figure 1). The majority of members in S5 (*AtMYB115*, *AtMYB119*, etc.) were involved in the regulation of glucosinolate biosynthesis. These natural chemicals likely enhance plant defenses against pests and confer the characteristic bitter flavor property in cruciferous vegetables [59]. Members of S15 (*AtMYB15*, *AtMYB17*, *AtMYB28*, etc.) play roles in lignin biosynthesis and stress resistance [60,61,62]. The expansion or contraction of *PebMYB* genes may result from asymmetric gene duplication events in different subgroups [63].

Furthermore, certain *PebMYB* genes (*PebMYB45*, *PebMYB57*, *PebMYB47*, *PebMYB13*, etc.) may also be involved in the response to drought stress based on gene homology analysis and functional characterization. This finding was also confirmed by qRT-PCR analysis, showing that seven *PebMYB* genes were associated with the drought stress response (Figure 7D–J). Notably, the similar functions of the *PebMYB* genes and their homologous genes in *A. thaliana* were found in other species as well. For example, *AtMYB60* and its ortholog in grape (*Vitis vinifera*), *VvMYB60*, also respond to drought stress, salt stress, and ABA treatment [64]. The homologous gene of *AtMYB15* in *Chrysanthemum morifolium* (*CmMYB15*) was associated with biotic stress resistance [65].

Gene duplication events, such as whole-genome duplication, tandem duplication, and segmental duplication, are major sources of new gene formation and functional diversity, playing a crucial role in evolution [66]. In this study, we screened duplication events and found that 176 *PebMYB* genes were derived from segmental duplication. Remarkably, these genes accounted for 76.5% of the total *PebMYB* genes and a mere 1.03% of the total duplicated genes (17,026 genes). This finding suggested that segmental duplications likely contributed to the diversification and expansion of *PebMYB* genes under purifying selection, which was also observed in the study of pearl millet [67]. Interestingly, we found that only three *PebMYB* genes were derived from tandem duplication, suggesting a relatively limited role of tandem duplication in the expansion of MYB genes in *P. eburnea* that was also observed in soybean and tobacco [68,69].

## 4. Materials and Methods

### 4.1. Identification of MYB Members

The whole genome sequences and protein sequences of *P. eburnea* were retrieved from a previous study [70]. A total of 198 sequences of *Arabidopsis* MYB genes (including 126 2R-MYB, 64 1R-MYB, five 3R-MYB, and three 4R-MYB) were downloaded from TAIR (www.arabidopsis.org, accessed on 25 May 2023) according to a previous study [16]. The *Arabidopsis* protein sequences were used as queries in BLAST v2.13.0 to identify *P. eburnea* MYB candidates with an e-value ≤ 1 × 10^−5^ and a bit score ≥ 100 [10,71]. The conserved domains of each candidate were confirmed in the InterPro database (www.ebi.ac.uk/interpro, accessed on 5 June 2023), while members lacking MYB domains were eliminated [72]. The Mw and pI of the proteins were determined by the TBtools program and the Expasy online program (https://web.expasy.org/compute_pi, accessed on 15 June 2023) [73,74]. The locations of *PebMYB* genes were obtained from the annotation file and visualized in TBtools.

### 4.2. Sequence Alignment and Phylogenetic Analysis

The protein sequences of *PebMYB* genes and 198 *AtMYB* genes were divided into two distinct categories based on their types (R2R3-MYB genes and the remaining types) and then used for multiple sequence alignment by MAFFT v7.505 software with default parameters [75,76]. To reveal the relationship between the *PebMYB* and *AtMYB* genes, phylogenetic analysis using the maximum-likelihood (ML) method was performed by IQ-TREE v1.6.12 software with parameters of 1000 ultrafast bootstraps and automatic model selection [77]. The phylogenetic results were then visualized in the iTOL (http://itol.embl.de, accessed on 10 July 2023) online program [78].

### 4.3. Analysis of Gene Structure, Motif and Cis-Acting Elements

The online MEME program (https://meme-suite.org/meme/tools/meme, accessed on 25 July 2023) was used to investigate the conserved motifs in the MYB genes of *P. eburnea* with the following parameters: maximum number of motifs detected = 20 and number of repeats = any [79]. The annotation file of the *P. eburnea* genome was used to define the gene structure, and the *PebMYB* protein sequences were used to construct an ML tree with the aforementioned method.

The promoter sequences of *PebMYB* genes (2000 bp upstream of the start codon) were extracted from the genome sequences according to the annotation file. The sequences were used to search for potential *cis*-acting elements in the PlantCARE online program (http://bioinformatics.psb.ugent.be/webtools/plantcare/html/, accessed on 5 September 2023). These elements were grouped into four groups based on their functions.

### 4.4. Gene Duplication and Synteny Analysis of PebMYB Genes

A BLASTp search was performed using 230 *PebMYB* protein sequences as queries against all protein sequences of *P. eburnea* with an e-value cutoff of 1 × 10^−5^. The collinearity relationship of the genome was calculated using MCScanX software according to the BLAST results and the genome annotation results [80]. The segmentally duplicated genes, gene density, and location information in the given annotated file were then visualized in Circos v0.69.9, while genes situated on unanchored scaffolds were omitted [81]. The Ka/Ks were calculated in TBtools based on the collinearity results of MCScanX analysis. The collinearity results between *P. eburnea* and other species were analyzed in MCScanX and visualized with TBtools.

### 4.5. RNA-seq Data Analysis and Network Construction

The RNA-seq data of four tissues (bud leaf, mature leaf, root, and stem) in *P. eburnea* were retrieved from a previous study [70]. After mapping reads to the genome of *P. eburnea* in HISAT2, the FPKM values of each gene were generated [82]. The FPKM values were normalized with the Z score method and visualized by the pheatmap package of R v4.2.2 to generate a heatmap.

For interacting network analysis, all the TFs with an FPKM greater than 1 in any of the tissues were selected for calculating the Pearson correlation coefficient (PCC). Only the absolute value of PCC greater than 0.95 was considered a potential interaction. The interacting network was constructed by Cytoscape v3.10.0 [83].

### 4.6. Drought Stress Treatment and qRT-PCR

For drought stress treatment, the seeds of *P. eburnea* were germinated in plastic pots with soil in a chamber at 25 °C and relative humidity at 70%. To ensure the uniformity of seedlings, every two uniform seedlings were transplanted to a pot filled with the same amount of soil and grown at Lushan Botanical Garden, Chinese Academy of Sciences, Nanchang, Jiangxi Province, China (115.8382° E; 28.9112° N). Six pots were divided into two groups (control and treatment). All plants were irrigated equally before treatment until they reached the 4-leaf stage. Drought stress was induced by ceasing irrigation for the treatment group, while seedlings in the control group were irrigated normally. Seedling leaves were sampled at three specific time points: before the initiation of drought stress treatment (stage 0, ST0) and after one month (stage 1, ST1) and two months (stage 2, ST2) of exposure to drought stress treatment.

For qRT-PCR analysis, total RNA was extracted from samples using an *EASYspin* Plus Complex Plant RNA kit (Aidlab Biotech, Beijing, China) according to the manufacturer’s instructions. First-strand cDNA was synthesized from 1 µg RNA with One-Step gDNA Removal and cDNA Synthesis SuperMix (TransGen, Beijing, China). qRTC-PCR was performed with PerfectStart Green qPCR SuperMix (TransGen, Beijing, China) on a CFX Connect Real-Time System (Bio-Rad, Hercules, CA, USA). The expression levels of the selected *PebMYB* genes were determined according to the comparative CT (2^−ΔΔCt^) method [84]. The *P. eburnea* glyceraldehyde-3-phosphate dehydrogenase (*PebGAPDH1*) was used as the internal reference gene. All qRT-PCRs were performed with three biological and three technical replicates. All primer sequences were designed by Primer Premier 5.0 and are listed in Supplementary Table S9.

## 5. Conclusions

In this study, a total of 230 *PebMYB* genes were identified in *P. eburnea*, including 67 1R-MYB, 155 R2R3-MYB, six 3R-MYB, and two 4R-MYB genes. A comprehensive analysis was performed to investigate phylogenetic relationships, motifs, gene structure, and synteny in *P. eburnea*. The prevalent purifying selection observed in the evolution of the *PebMYB* genes highlights their functional conservation based on the Ka/Ks results. Further analysis of the expression of the *PebMYB* genes in four different tissues revealed that they could be divided into five groups based on their expression characteristics. The expression profiles were verified by qRT-PCR analysis. Importantly, the identification of *PebMYB* genes that were homologous to drought-responsive genes in *A. thaliana*, as well as the characterization of their expression under drought stress, broadened our understanding of their potential roles in drought adaptation. The seven genes (*PebMYB3*, *PebMYB13*, *PebMYB17*, *PebMYB51*, *PebMYB142*, *PebMYB69*, and *PebMYB95*) showed significant differences between the control and drought treatments, suggesting that they are likely involved in the drought stress response in *P. eburnea*.

Our findings shed a basis on understanding MYB genes in *P. eburnea* and provide valuable insights for future studies investigating the molecular mechanisms underlying drought stress. Moreover, these results serve as a valuable resource for future genetic engineering and breeding programs aimed at improving the drought tolerance of *P. eburnea* and related species.

## Data Availability

The transcriptomic sequencing data can be downloaded in the NCBI Sequence Read Archive under accession number PRJNA934730.

## Supplementary Materials

The following supporting information can be downloaded at: https://www.mdpi.com/article/10.3390/ijms25010465/s1.
