# Supplementary material for: Genome-Wide Analysis of MYB Genes in Primulina eburnea (Hance) and Identification of Members in Response to Drought Stress
Source: Int J Mol Sci. 2023 Dec 29;25(1):465. doi: 10.3390/ijms25010465 (PMC10778706; doi:10.3390/ijms25010465)
Supplement: Supplementary file 1 [file ijms-25-00465-s001.zip › 20231026pebmyb-SF_ijms_type.pdf]

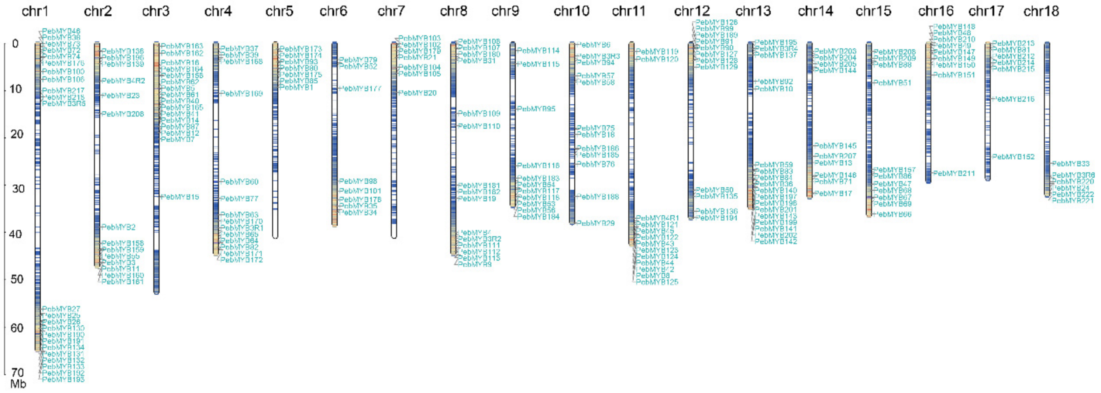

Figure S1. The location of *PebMYB* genes.

The *PebMYB* genes on the 18 chromosomes are represented in cyan color where the gene density was showed by heatmap in 18 chromosomes. The scale bar is 10 Mb.

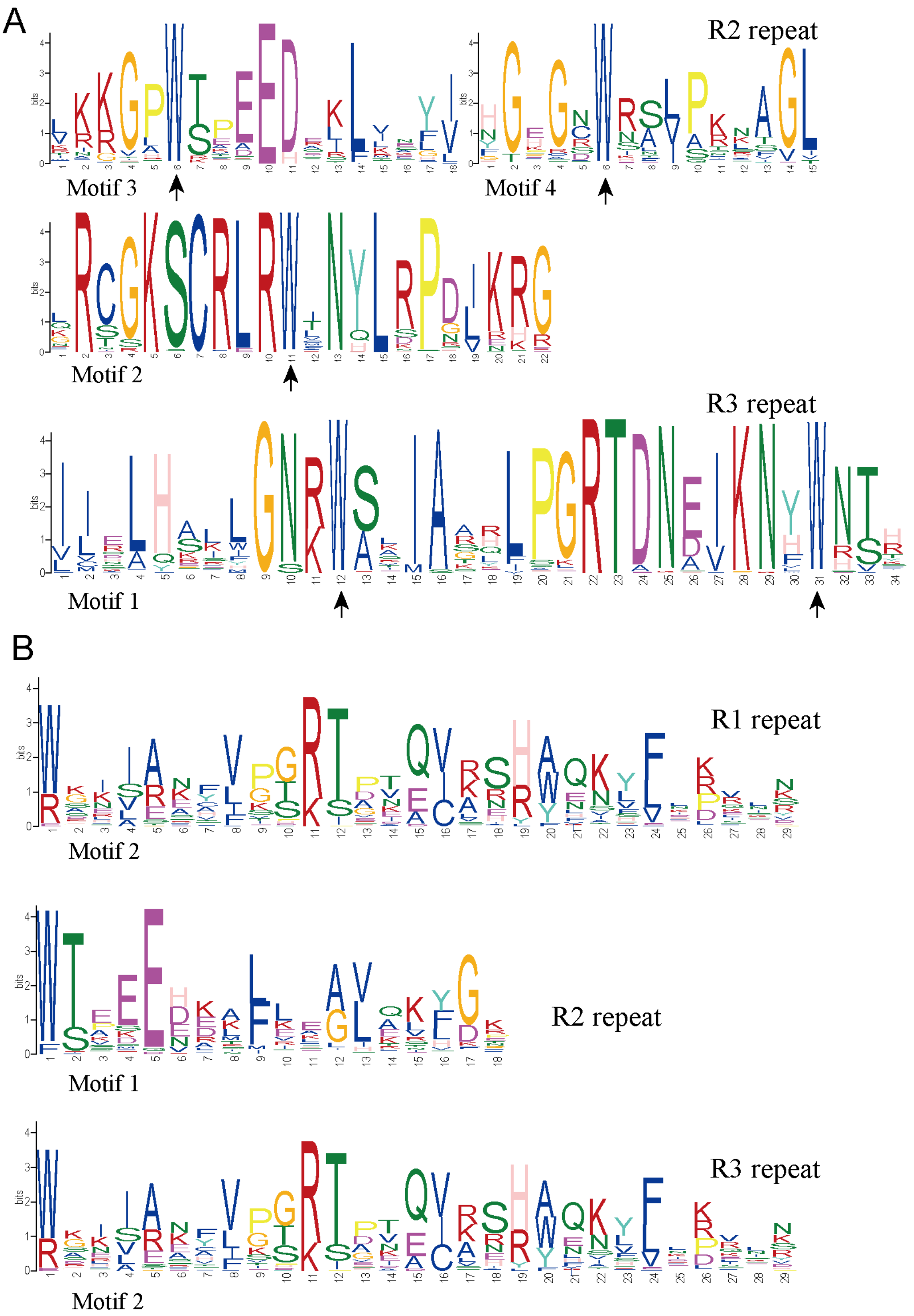

Figure S2. The seq-log of MYB domains in *P. eburnea*.

A. The R2 and R3 repeats of R2R3- MYB genes. The arrows indicated the typically conserved tryptophan (W) residues. **B.** The R1, R2 and R3 MYB repeats of 1R-MYB, 3R-MYB, 4R-MYB. The overall height of each stack indicated the conservation of the MYB protein sequence at the point. Letters indicated the different type of amino acid residue.

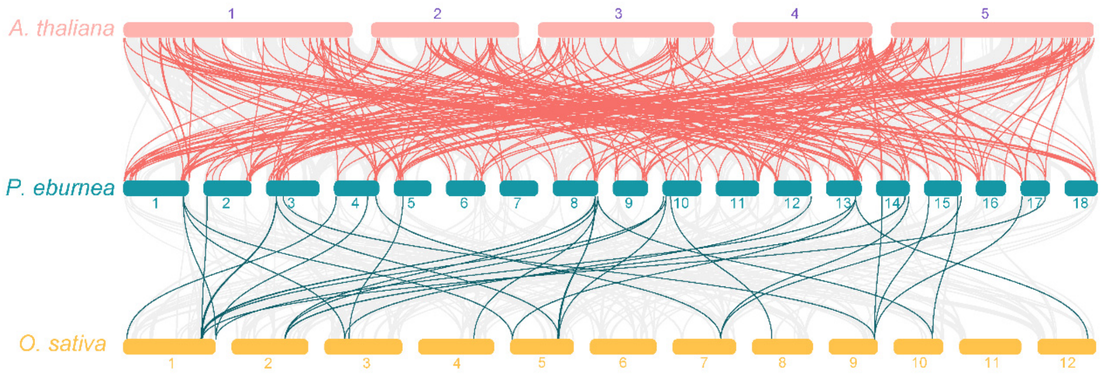

Figure S3. The synteny results of *PebMYB* genes.

Synteny analysis between P. eburnea and other two species. The collinear genes between species were marked in grey while the collinear PebMYB genes were marked in salmon or cyan color.

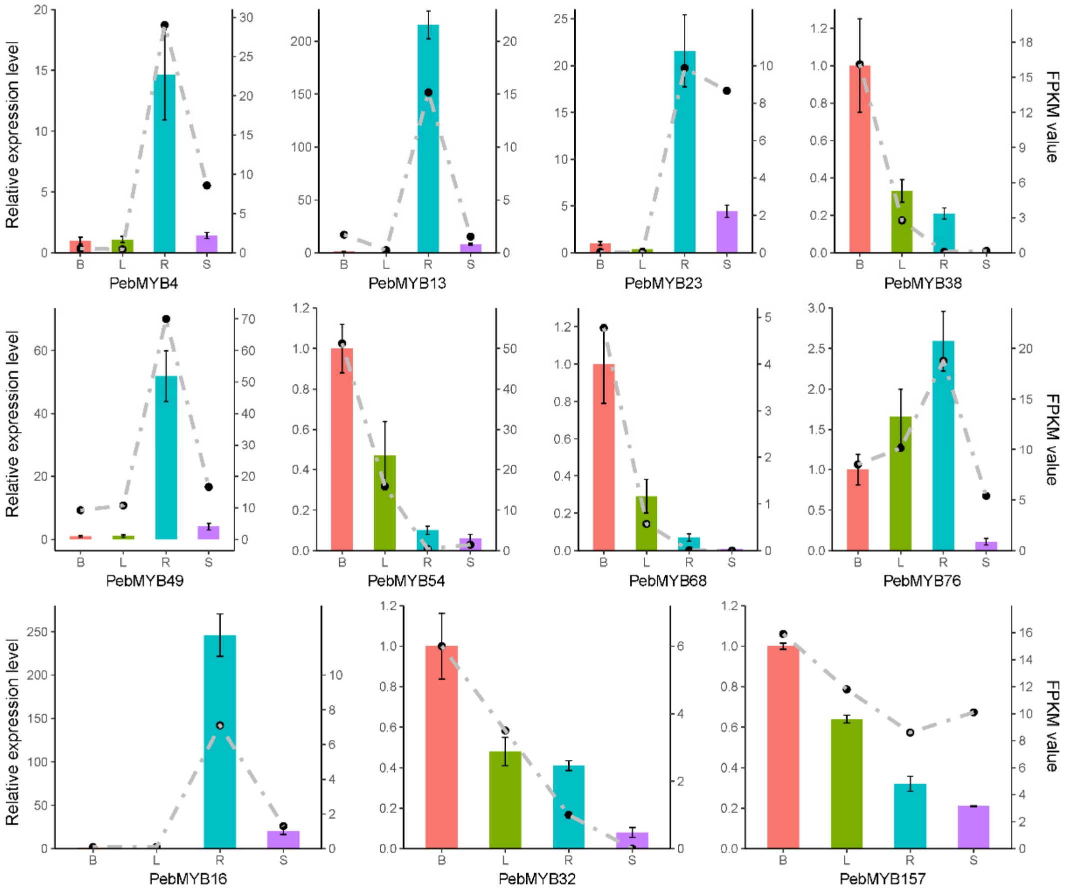

Figure S4. Validation of RNA-seq data by qRT-PCR

The left y-axis (corresponding to the columns) indicates the relative expression levels in qRT-PCR, whereas columns and bars indicate the mean value and standard error. The right y-axis (corresponding to the black dots and grey dotted lines) indicates FPKM value of RNA-seq.

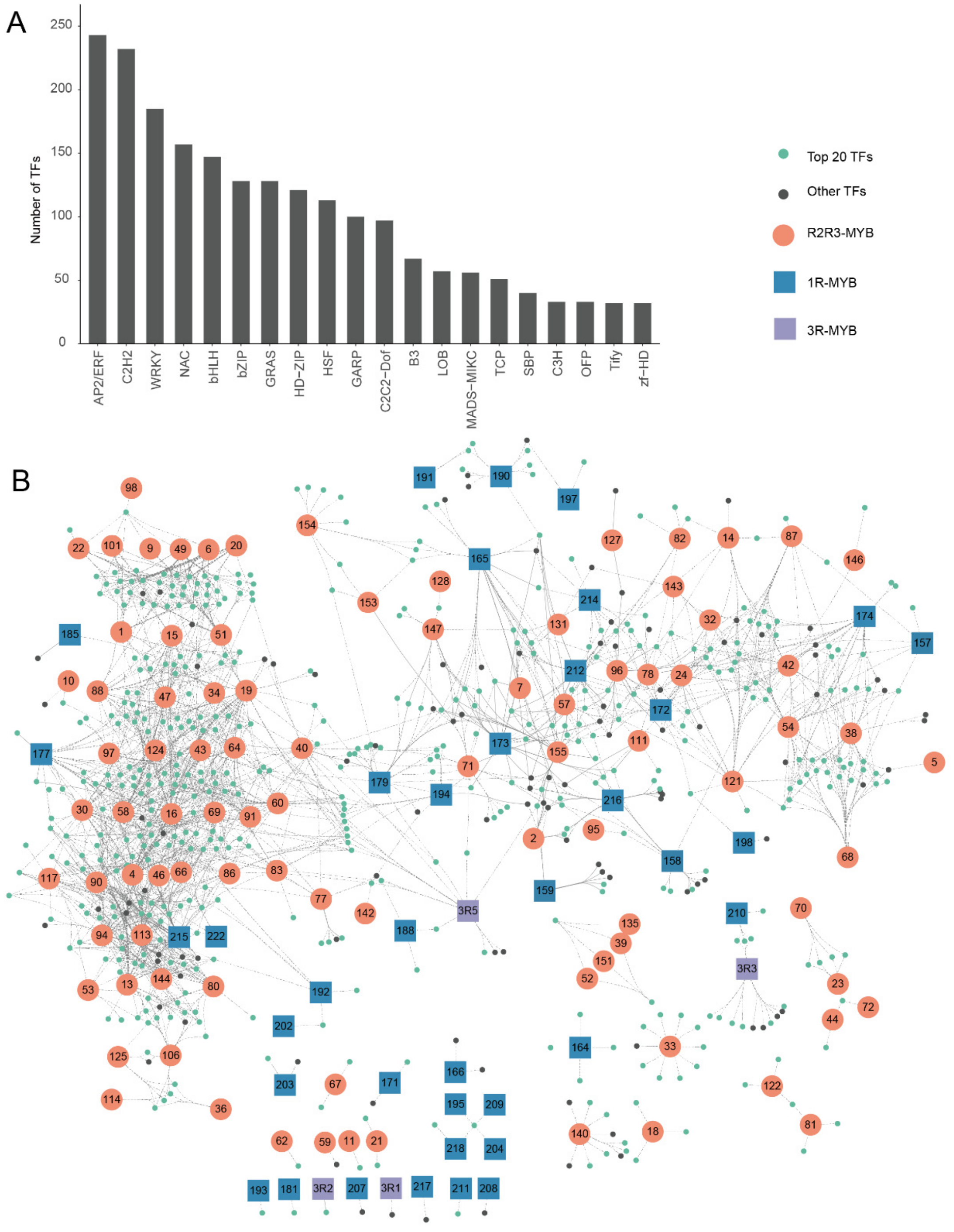

Figure S5. Interactions between *PebMYB* genes and other transcription factors (TFs).

A. The top 20 TF families with the highest number of members co-expressed with PebMYB genes. **B.** the potential interacting network between PebMYB genes and other TFs. Solid lines indicate positive interaction while dash lines indicate negative interaction. The corresponding PebMYB genes were indicated by character or number.
